# Chronic Post-Traumatic Aortic Isthmus Pseudoaneurysm After Conservative Management of Grade II Injury: Why Is Continuous Follow-Up Mandatory?

**DOI:** 10.3390/jcm14041133

**Published:** 2025-02-10

**Authors:** Simona Sica, Giovanni Tinelli, Ottavia Borghese, Manav Dimri, May Dvir, Fabrizio Minelli, Antonio Rizza, Piergiorgio Bruno, Massimo Massetti, Yamume Tshomba

**Affiliations:** 1 Faculty of Medicine and Surgery, Università Cattolica del Sacro Cuore, 00168 Rome, Italy; giovanni.tinelli@unicatt.it (G.T.); ottaviaborghese@gmail.com (O.B.); manav.dimri01@icatt.it (M.D.); maydvir2@gmail.com (M.D.); fabrizio.minelli@unicatt.it (F.M.); piergiorgio.bruno@policlinicogemelli.it (P.B.); massimo.massetti@policlinicogemelli.it (M.M.); yamume.tshomba@unicatt.it (Y.T.); 2 Unit of Vascular Surgery, Fondazione Policlinico Universitario A. Gemelli-IRCCS, 00168 Rome, Italy; 3 Unit of Cardiology, Ospedale del Cuore, Fondazione Toscana “G. Monasterio”, 56100 Massa, Italy; antonio.rizza@ftgm.it; 4 Unit of Cardiac Surgery, Fondazione Policlinico Universitario A. Gemelli-IRCCS, 00168 Rome, Italy

**Keywords:** blunt thoracic aortic injuries, pseudoaneurysm, trauma, TEVAR, follow-up

## Abstract

**Background:** Grade I-II blunt traumatic aortic injuries (BTAIs) are typically managed conservatively, but their long-term progression is poorly understood. Chronic pseudoaneurysms may develop years after the injury, often remaining asymptomatic and being incidentally diagnosed. **Methods:** Two cases of post-traumatic aortic pseudoaneurysms, detected 20 and 25 years following conservatively managed BTAIs, are reported. Additionally, a comprehensive review of all post-traumatic pseudoaneurysms reported in the MedLine (PubMed.gov, U.S. National Library of Medicine, National Institute of Health) database between January 1984 and December 2024 was performed. **Results:** Both our patients underwent successful hybrid procedures, with no complications at the 1- and 4-year follow-ups. Our literature review identified 37 patients across 22 studies, with 32.4% presenting asymptomatically or incidentally diagnosed through routine imaging, between 1 month and 50 years after the initial trauma. In 37.8% of cases, the patients underwent open or endovascular repair. **Conclusions:** This case series and literature review emphasize the importance of long-term follow-up for patients with conservatively managed BTAIs, as chronic complications such as aortic pseudoaneurysms can arise decades later. Continuous surveillance is critical to ensure early detection and management.

## 1. Introduction

Blunt traumatic aortic injuries (BTAIs) are rare but potentially fatal consequences of blunt trauma, occurring in 0.3–1% of cases [1]. Grade III (pseudoaneurysm) and IV (rupture) BTAIs represent more than a third of cases and require immediate interventions, while conservative management could be performed for less extensive lesions [2]. However, there is uncertainty regarding whether grade II (intramural hematoma) BTAIs should be managed with endovascular or surgical repair or nonoperative measures. Nevertheless, untreated injuries may evolve into chronic aortic pseudoaneurysms, which occur in 2–5% of cases and are often incidentally discovered even years after the acute onset [3,4]. Despite their rarity, aortic pseudoaneurysms present a significant risk due to their high potential for rupture, making timely diagnosis and management critical [5].

In this paper, we report a case series of two patients who were treated at our institution (Fondazione Policlinico Universitario A. Gemelli-IRCCS, Rome, Italy) for chronic post-traumatic pseudoaneurysms of the aortic arch, detected 20 and 25 years after grade II BTAI, respectively. Written informed consent was obtained from the patients for publication of their case and accompanying images.

A comprehensive literature review of reported cases of post-traumatic pseudoaneurysm published on the MedLine (PubMed.gov, U.S. National Library of Medicine, National Institute of Health) database between 1 January 1984 and 31 December 2024 was also performed.

## 2. Cases

### 2.1. Case 1

A 48-year-old man with a significant past medical history of eye prosthesis placement due to glioma and previous surgeries for tonsillectomy and hemorrhoidectomy presented to our institution in February 2023 with a three-month history of persistent cough. His medical history was further notable for a car accident 25 years earlier, which resulted in a grade II BTAI that had been managed conservatively without follow-up. Given the persistent cough, which was unresponsive to medical treatment, and the absence of signs of pneumopathy, an instrumental exam was performed.

**Figure 1:**
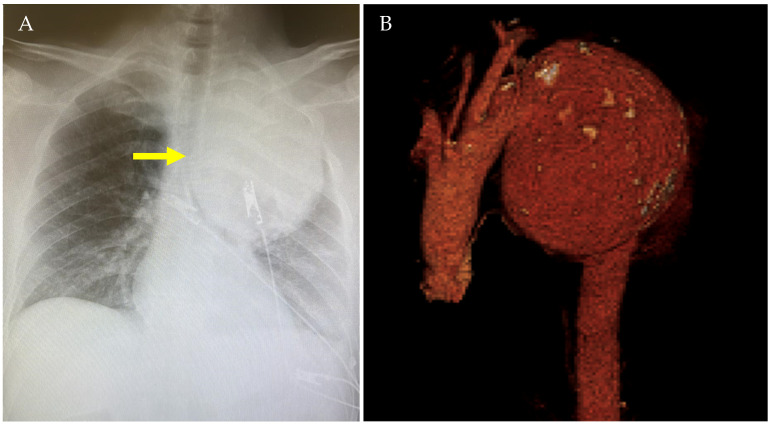
Chest radiography (**A**) and three-dimensional volume-rendered reconstruction of the pre-operative CTA (**B**) showing a 11.5 cm sacciform aneurysm of the distal aortic arch (yellow arrow).

The chest radiography revealed the presence of a 13 × 11 cm coarse oval neoformation at the level of the upper lobe of the left lung. Given the abnormal radiograph findings, a computed tomography angiography (CTA) was performed. The CTA showed a sacciform aneurysm of the distal aortic arch with 11.5 cm in maximal diameter (Figure 1).

The patient was admitted to the hospital for further evaluations in hemodynamically stable conditions. Initial investigations included an echocardiogram, which revealed left ventricular hypertrophy, with a normal left ventricular ejection fraction (60%). Further imaging with a CT coronary angiogram identified moderate stenosis of the left anterior descending coronary artery, not requiring intervention.

**Figure 2:**
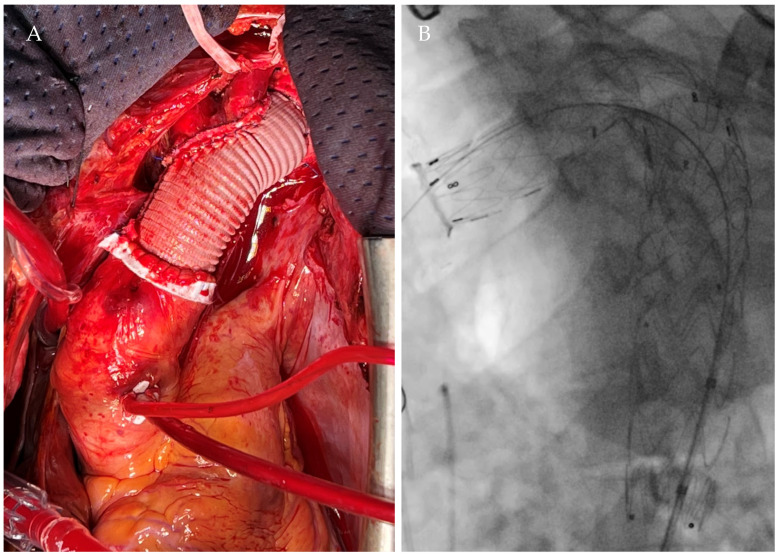
Elephant Trunk procedure (**A**) and endovascular (**B**) aneurysm exclusion.

Treatment options were discussed in a multidisciplinary team. Given the age, comorbidities, and anatomical characteristics, a hybrid approach with aortic arch open repair and thoracic endovascular aortic repair (TEVAR) was proposed. The procedure was performed under general anesthesia, in a hybrid operating room and under fusion imaging guidance. After full sternotomy and extracorporeal circulation, the cardiac team performed the Elephant Trunk (ET) procedure with the use of a 24 mm vascular Dacron graft (Gelweave, Terumo Aortic, Sunrise, FL, USA). Left carotid-subclavian bypass with an 8 mm Dacron graft (Gelweave, Terumo Aortic, Sunrise, FL, USA) with end-to-side proximal and distal anastomosis using a 5/0 polypropylene running suture and reimplantation of the brachiocephalic trunk and left common carotid artery were performed. The endovascular step was performed through a bilateral femoral percutaneous approach. The 32-32-185 mm Valiant Captivia (Medtronic Inc., Santa Rosa, CA, USA) stent graft was deployed in the ET prosthesis under rapid pacing, followed by the deployment of the second stent graft 32-32-150 Valiant Captivia (Medtronic Inc., Santa Rosa, CA, USA) (Figure 2).

The completion angiography demonstrated the patency of the supra-aortic trunks, the correct positioning of the stent graft, and complete exclusion of the aortic pseudoaneurysm without endoleaks.

The post-operative course was uneventful, with a one-day stay in the intensive care unit after the index procedure; the patient was discharged home on post-operative day 8. The CTA that was performed on post-operative day 7 showed no aortic-related complications.

**Figure 3:**
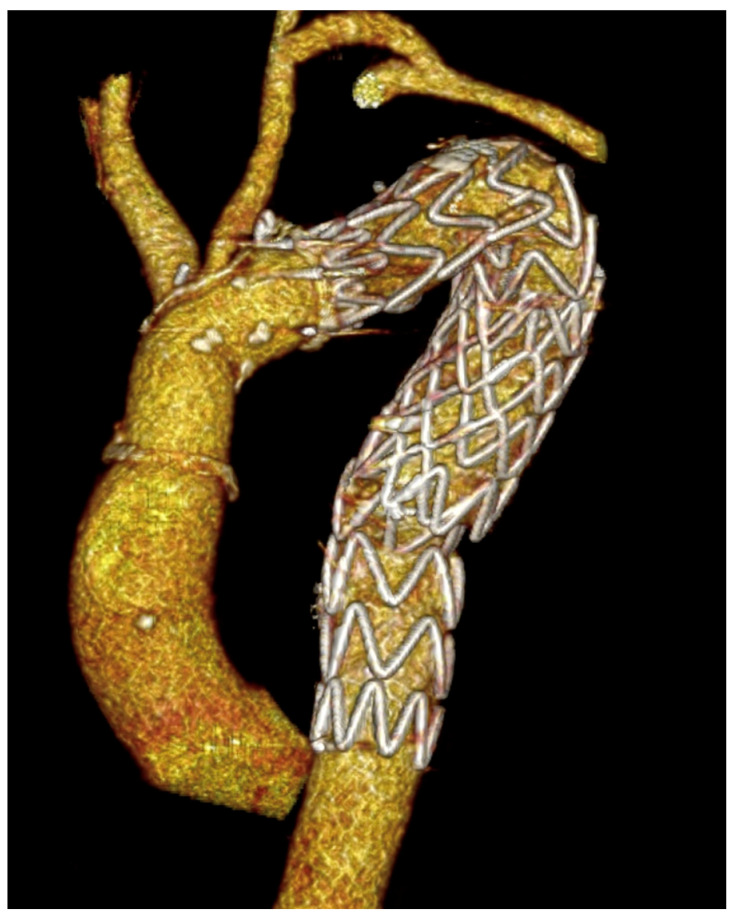
Three-dimensional volume-rendered reconstruction of the 1-year CTA.

At the 1-year follow-up, the patient was doing well, and the CTA showed the patency of the stent graft and all supra-aortic trunks without any endoleaks (Figure 3).

### 2.2. Case 2

**Figure 4:**
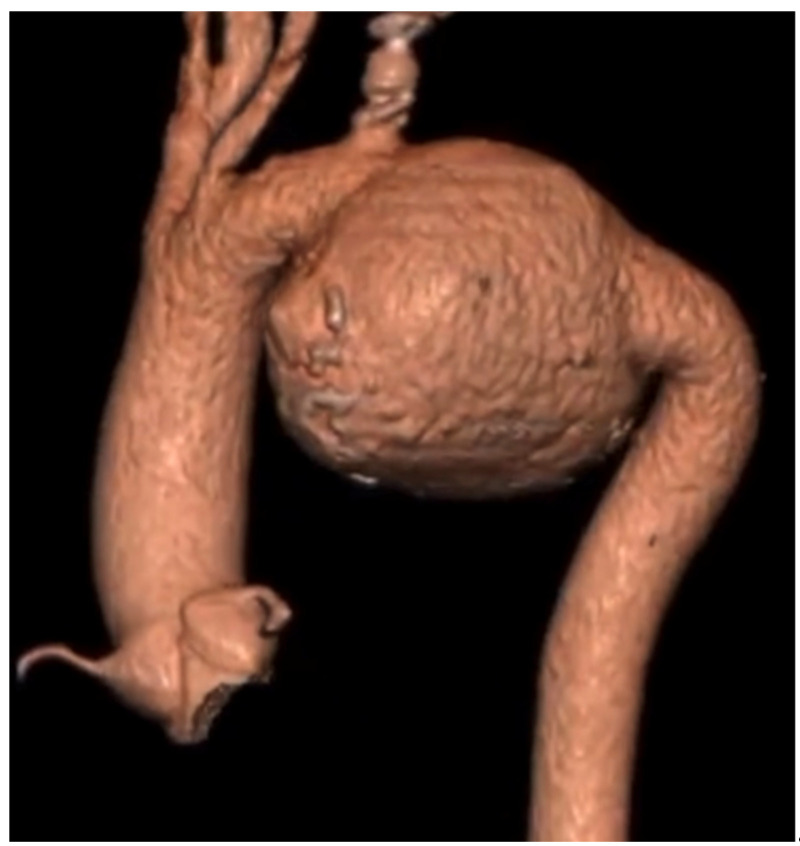
Three-dimensional volume-rendered reconstruction of the pre-operative CTA showing the 8.5 cm aortic pseudoaneurysm.

A 46-year-old male patient was electively admitted to our institution in May 2020 for the incidental diagnosis of an 85 mm maximal diameter aortic isthmus pseudoaneurysm, discovered on a CTA that was performed for pneumological screening (Figure 4). No symptoms or signs of the pseudoaneurysm were observed. His medical history included chronic obstructive pulmonary disease (COPD) with mild restrictive lung deficiency, hypertension, dyslipidemia, smoking (15 cigarettes/day), obesity (BMI 31), and hyperuricemia. The patient had been involved in a polytraumatic incident 21 years earlier, resulting in a grade II thoracic aortic injury that was conservatively managed. However, he was lost to follow-up after that incident.

Treatment options were discussed in a multidisciplinary team. Given the age, comorbidities, anatomical characteristics, and patient refusal of total open arch repair, a hybrid approach with a carotid-subclavian bypass and custom-made scalloped stent graft for the left carotid artery was proposed. Waiting for the stent graft customization of 3 weeks, the left carotid-subclavian bypass and left subclavian embolization with a 14 mm Amplatzer Plug II (Abbott Vascular, Redwood City, Calif) were performed.

The TEVAR procedure was performed under general anesthesia, in a hybrid operating room setting and under redo fusion imaging guidance (in addition to the bone, the previous plug was used as a marker to allow for more accurate fusion imaging). Ultrasound-guided puncture was performed bilaterally on the common femoral arteries. The 30-26-180 scalloped device (Bolton Relay, Terumo Aortic, US) was deployed below the origin of the left common carotid artery in proximal landing zone 1, with rapid pacing.

**Figure 5:**
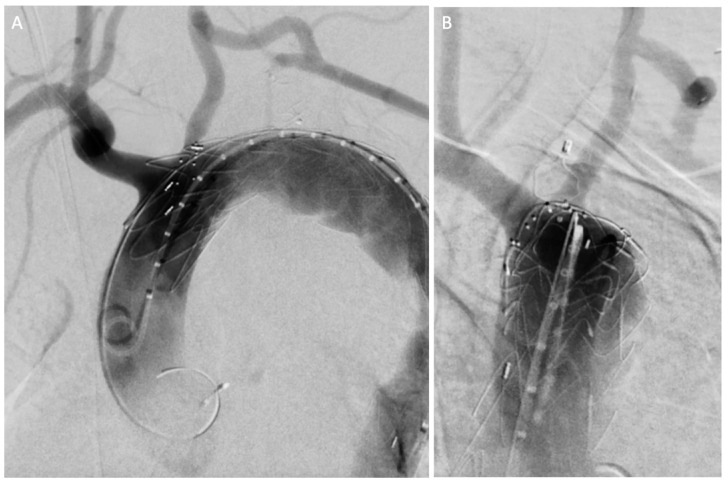
Final angiography showing the correct position of the stent graft and the patency of the supra-aortic trunks without endoleaks in anteroposterior (**A**) and right anterior oblique (**B**) projections.

The final angiography (Figure 5) and cone-beam computed tomography showed the correct position of the graft and patency of all supra-aortic trunks. The complete endovascular aortic repair is summarized in Video S1.

The post-operative CTA showed the correct positioning of the endograft without endoleaks, as well as the patency of the graft and all supra-aortic trunks. After an unremarkable clinical course, the patient was discharged in good clinical condition on post-operative day 4.

**Figure 6:**
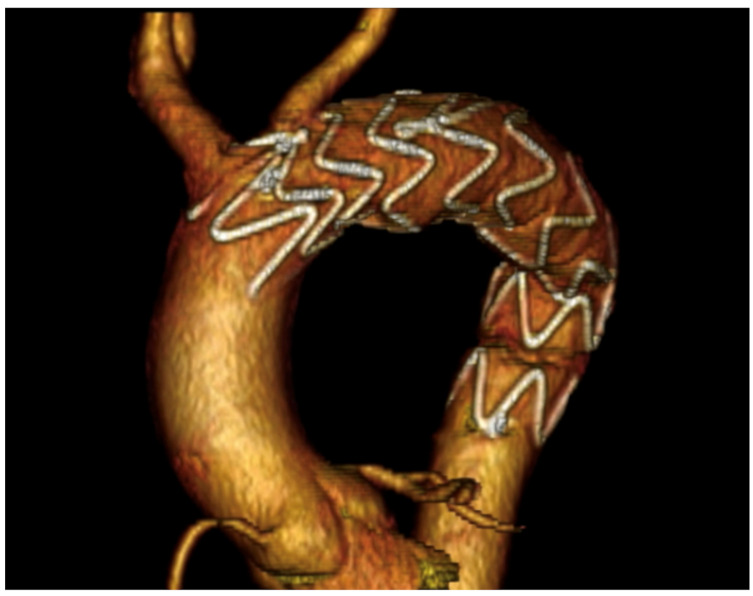
Three-dimensional volume-rendered reconstruction of the post-operative 4-year CTA.

Four years after surgery, the patient was doing well, with no aortic-related complication and a technical success at the follow-up CTA (Figure 6).

## 3. Literature Review

A literature review was conducted through the MedLine database (PubMed.gov, U.S. National Library of Medicine, National Institutes of Health) from January 1984 to December 2024, focusing on manuscripts reporting chronic aortic pseudoaneurysms following BTAIs. The search strategy involved a combination of the following terms: “TEVAR”, “BTAI”, “aortic trauma”, “chronic pseudoaneurysm”, and “blunt traumatic aortic injury”. Only articles published in English with full-text access were included. The selected papers had to report on the clinical presentation, timing, and treatment strategies of chronic aortic pseudoaneurysms associated with BTAI. Titles and abstracts were reviewed for relevance, and data were extracted using a predefined database. The initial search identified 189 articles, of which 167 were excluded due to a lack of clinical data or differing focus. The remaining studies were evaluated for risk of bias according to the CARE guidelines and the Joanna Briggs Institute (JBI) Critical Appraisal Checklist for Case Reports/Studies and Case Series, as only case reports and case series were included. Due to the heterogeneity of the reported data, a systematic review or meta-analysis of the pooled clinical results was not possible, and only a narrative synthesis is provided.

Twenty-two papers were included, totaling 37 patients (n = 37), including the 2 cases presented above. Details of included cases papers are summarized in Table 1. The majority of patients were male (n = 28, 75.7%), with ages ranging from 19 to 76 years. In 73% of cases, the blunt trauma involved the descending thoracic aorta (DTA). In 32.4% of cases, the patients were asymptomatic with an incidental diagnosis at routine chest radiography.

**Table 1:** Details of included cases papers.

Author	Age (y)	Sex	Location of the Pseudoaneurysm	Symptoms	Interval from BTAI to Pseudoaneurysm Diagnosis	Treatment
Moore EH et al. [6]	30	M	Proximal DTA	Asymptomatic (incidentally discovered on routine chest radiography)	5 years	Open surgery
Ferrante SL et al. [7]	28	M	Aortic isthmus	NR	8 years	Open surgery
McNamee CJ et al. [8]	33	F	Aortic isthmus	Symptomatic (unspecified)	3 months	Open surgery
Albuquerque F et al. [9]	34	M	Ascending aorta	Asymptomatic	12 years	Open surgery
Bacharach JM et al. [10]	(1) 31 (2) 27	(1) F (2) F	(1) Aortic isthmus (2) Distal arch and proximal DTA	(1) Asymptomatic (2) Asymptomatic	(1) 16 years (2) 9 years	(1) NR (2) Conservative
Fidvi SA et al. [11]	48	M	Proximal DTA	Bilateral lower-extremity cellulitis	31 years	NR
Cartier R et al. [12]	NR	F	Thoracic and abdominal aortic	NR	10 years	TEVAR
Saltman AE et al. [13]	43	M	DTA	Chest pain	15 years	Refused treatment
Rousseau H et al. [14]	14–76 Mean 37	8 M, 1 F	Aortic isthmus	NR	1 month–32 years Mean 10 years	TEVAR
Kaminishi Y el al [15]	(1) 40 (2) 57	(1) M (2) M	Aortic isthmus	(1) not mentioned (2) 3-month history of hoarseness	(1) 24 years (2) 17 years	(1) Open surgery (2) Open surgery
Gawenda M et al. [16]	NR	NR	Aortic isthmus	Asymptomatic (incidentally discovered on routine chest radiography)	NR	TEVAR
Deng YB et al. [17]	28	M	Ascending aorta	Dyspnea and peripheral edema	1 year	Open surgery
Gurkan S et al. [18]	28	M	DTA	5 months persistent coughing	6 months	Open surgery
Bizzarri F et al. [3]	33	M	DTA	Back chest pain and cough	4 years	TEVAR
Fatimi SH et al. [19]	50	M	Aortic isthmus	4-month history of non-productive cough	15 years	Open surgery
Marcu CB et al. [20]	59	M	Aortic isthmus	New-onset angina pectoris	30 years	Conservative
Ghaffari S et al. [21]	19	M	DTA	4-month history of non-productive cough	8 months	Open surgery
Pozek et al. [5]	75	M	DTA	Asymptomatic (incidentally discovered on routine chest radiography)	50 years	NR
Nizet C et al. [22]	34	M	Aortic isthmus	Dysphagia	10 years	Planned debranching procedure + TEVAR, but due to rupture during the procedure, TAR was performed
Pourafkari et al. [23]	52	F	Ascending aorta	Asymptomatic	2 years	Open surgery
Karangelis D et al. [24]	48	M	Aortic isthmus	Asymptomatic (incidentally discovered on routine chest radiography)	20 years	Open surgery
Azizi Z et al. [25]	29	F	Aortic arch	Sinus tachycardia, and cough (compressive effect)	2 years	Open surgery
Al-Adawi et al. [26]	(1) 37 (2) 37 (3) 29	(1) M (2) F (3) M	(1) Proximal DTA (2) Aortic isthmus (3) DTA	(1) Asymptomatic (2) Asymptomatic (3) Asymptomatic	(1) 18 years (2) 9 years (3) 6 years	(1) TEVAR (2) TEVAR (3) Refused treatment
Current Case-1	48	M	Aortic arch	Cough	25 years	Hybrid (ET + TEVAR)
Current Case-2	46	M	Aortic arch	Asymptomatic	21 years	Hybrid (carotid-subclavian bypass + TEVAR)

DTA—descending thoracic aorta; TAR—total arch replacement; TEVAR—thoracic endovascular aortic repair; ET—Elephant Trunk, NR—not reported; M—male; F—female; y—years.

The aortic pseudoaneurysm was detected between 1 month and 50 years after the initial trauma, mostly incidentally during imaging for other comorbidities.

The clinical characteristics are summarized in Table 2. Treatment was mostly in-terventional (including endovascular or open repair), and only three cases were man-aged conservatively due to refusal of the patients. Follow-up data about clinical out-comes and early and long-term survival were not available.

## 4. Discussion

BTAI is the second most common cause of death in trauma patients that occurs in 80% of cases before hospitalization [27]. Deceleration during high-velocity motor vehicle accidents is the most frequent underlying cause, followed by crushing accidents, falls from a height, and other deceleration accidents that occur during sports [1]. The aortic isthmus, being the site of transition from the unfixed aortic arch to the fixed descending thoracic aorta and the relatively lesser tensile strength of this region, is the most affected segment on radiological findings. Indeed, during deceleration trauma, the displacement of the aorta in a cranial or caudal direction causes tears at the isthmus because of the traction exercised by surroundings (the ligamentum arteriosum, the left main stem bronchus, and the paired intercostal arteries) that are fixed. Lesions of the proximal ascending aorta (8–27%) and the aortic arch (8–18%) are, conversely, more frequently immediately lethal and mostly reported in autopsy series [27]. When the abdominal aorta is affected, the aortic injury is frequently associated with other vascular lesions affecting the inferior mesenteric artery (33%) or the renal arteries (24%) [28].

The Advanced Trauma Life Support principles recommend performing chest radiograph in the majority of trauma patients, and it has been reported that BTAI may be firstly diagnosed on chest radiography (in 41–89% of cases) due to the evidence of several signs of a widened mediastinum, obliteration of the aortic contour, loss of the paravertebral pleural stripe, displacement of the left mainstem bronchus, deviation of the nasogastric tube, and left apical cap, as well as the severity and extension of the underlining aortic lesions. Trans-esophageal echocardiography allows for the visualization of the proximal and transverse aorta, but diagnosis should always be confirmed with CTA, which provides detailed data about the aortic anatomy and allows for a proper decision on optimal management strategies and preprocedural planning [29].

Aortic lesions due to trauma may be identified using several classifications (i.e., the historical Parmley’s classification or the “University of Washington (UW) revised criteria”), but the most applied is from the Society for Vascular Surgery (SVS) trauma. According to the SVS classification, BTAIs are divided into four grades (grade I: intimal tear; grade II: intramural hematoma; grade III: aortic wall disruption with pseudoaneurysm; and grade IV: disruption of the aortic wall with free rupture) considering the extension and severity of aortic lesions at the radiological imaging, which intuitively correlate with the indication for surgical treatment [2].

Indeed, the current recommendations from the Society for Vascular Surgery only indicate clinical and imaging supervision for grade I injuries, as blood pressure control (systolic ≤ 100 mmHg, mean arterial pressure of ≤80 mmHg, and a heart rate of ≤100 beats per minute) seems to be sufficient to prevent the progression of this lesion, decreasing the risk of rupture from 12% to 1.5% [29]. Conversely, grade II injuries may be managed medically or by TEVAR, and grade III-IV BTAI should always be treated [30]. More specifically, patients presenting with grade IV lesions should undergo urgent repair, whereas for grade II-III lesions, delayed intervention (>24 h) seems to guarantee a significantly lower mortality rate (5.8% versus 16.5%) than immediate surgery, as it allows clinicians to optimize the patient’s clinical status by treating the associated injuries before the aortic procedure [30,31].

Overall, approximately one in four patients with BTAI will receive medical management as definitive therapy, as shown in a recent multicenter study by Arbabi and colleagues [32] including 432 trauma patients, but little is known regarding the natural history of those lesions if medically managed, since no recommendations currently exist about the optimal clinical and radiological follow-up. It is reported that the rate of chronic pseudoaneurysm evolution after BTAI ranges from 2 to 5% [4]. The outcome rates of SVS grade II blunt traumatic aortic injury when treated nonoperatively were estimated at 10.4% for all-cause mortality, 2.9% for aorta-related mortality, and 3.3% for early aortic intervention [33]. However, given that progression of the BTAI is possible, follow-up aortic imaging is encouraged, as well as appropriate blood pressure control and exercise restriction [33].

Patients with BTAIs tend to be young, while the present review demonstrated that chronic pseudoaneurysm could be detected in all age groups, as these complications may be incidentally diagnosed even years after the acute trauma, ranging between 3 months and 50 years. The signs and symptoms of chronic post-traumatic aortic pseudoaneurysms can range from asymptomatic to more severe presentations, including chest or back pain, shortness of breath, and, in some cases, rupture leading to hemorrhagic shock. Differential diagnosis should include other causes of aortic pathology, such as aneurysms due to atherosclerosis or infectious etiologies and dissection. For instance, we reported two cases of incidentally discovered aortic pseudoaneurysms that were detected more than two decades after a BTAI. This highlights that although compliance with follow-up can be challenging, life-long surveillance is essential. Following our experience and this review of the literature, we underline the importance of continuous follow-up, even in patients who were conservatively treated at the first encounter, to allow for prompt management and prevention of any further complications.

Because of their rarity, no standardized treatment pathway is currently available for chronic post-traumatic pseudoaneurysms. However, since the risk of rupture is high, an interventional treatment is often mandatory [6]. Several strategies have been adopted, including conservative management for small and asymptomatic lesions, endovascular, open repair, or hybrid strategies based on the individual anatomy and clinical presentation [34,35]. The present review showed that conservative management, which mostly includes blood pressure control, can rarely be carried out, and it is normally selected based on the patient’s unfitness or refusal of invasive management [20,25]. Hence, tailored interventional management is required in most cases.

The superiority of one treatment over another has not been proven yet: open repair may consist of total arch replacement or interposition graft according to the extension of the lesion. Currently, an important shift towards endovascular treatment can be clearly observed. The potential benefits of TEVAR are linked to the reduced invasiveness compared with open surgery [36,37,38,39,40]. A meta-analysis of 589 patients showed that endovascular treatment of descending thoracic aortic trauma is an alternative to open repair and is associated with a lower 30-day mortality (8% with TEVAR vs. 20% with open repair, *p* = 0.005) and ischemic spinal cord complication rates (0% with TEVAR vs. 7% with open repair, *p* = 0.03) [40]. However, complications such as endograft migration, endoleak, device fracture, and access complications could lead to reintervention during the long-term follow-up [36]. Indeed, TEVAR is a relatively new technology, of which the long-term durability remains unknown. The current review demonstrated that while multiple strategies are viable, there is a significant gap in data regarding survival rates and complications associated with each approach in case of a BTAI. Further investigation, especially focusing on long-term outcomes, is essential before recommending one strategy over another.

## 5. Conclusions

This case series highlights the importance of continuous follow-up in all patients with traumatic injuries involving the aorta, even if not requiring immediate surgical repair. While patients with BTAIs are typically young and have a long life expectancy, the progression of grade II BTAI over time has not been thoroughly investigated, and complications may be encountered even years after the acute onset. Chronic pseudoaneurysms may remain asymptomatic for decades, often presenting only with life-threatening events such as rupture.

Multidisciplinary protocols should be established for the successful management of BTAIs. Continuous follow-up of these patients is mandatory, as it could allow for the early diagnosis of complications, optimizing the clinical outcomes.

## 6. Limitations

This study is inherently limited by the non-standardized nature of the case reports that were selected for the analysis, impacting the uniformity of the variables of interest.

## Figures and Tables

**Table 2:** Clinical and technical summary of included papers.

	Number of Patients n/37 (%)
Sex *	
Male	28/36 (77.7)
Female	8/36 (22.2)
Age	Range 19–76 years
Location	
Ascending aorta	2 (5.4)
Aortic arch	8 (21.6)
Descending thoracic aorta	27 (73)
Clinical presentation (one of more of the following)	
Asymptomatic/incidentally discovered	12 (32.4)
Pain	3 (8.1)
Cough	6 (16.2)
Other or not reported	18 (48.6)
Management strategies	
Conservative	4 (10.8)
Endovascular repair	14 (37.8)
Open repair	14 (37.8)
Hybrid repair	2 (5.4)
Not reported	3 (8.1)

Data are reported as n (%) or mean/range. *: sex was not disclosed in one paper.

## Supplementary Materials

The following supporting information can be downloaded at https://www.mdpi.com/article/10.3390/jcm14041133/s1: Video S1: Step-by-step procedure of the 85 mm post-traumatic chronic pseudoaneurysm endovascular aortic repair.
